# Trends and patterns of maternal deaths from 2015 to 2019, associated factors and pregnancy outcomes in rural Lagos, Nigeria: a cross-sectional study

**DOI:** 10.11604/pamj.2023.44.185.37567

**Published:** 2023-04-20

**Authors:** Ibukun Abosede Oladipo, Margaret Omowaleola Akinwaare

**Affiliations:** 1 Department of Nursing, College of Medicine, University of Ibadan, Ibadan, Nigeria

**Keywords:** Maternal health, pregnancy, childbirth, Nigeria

## Abstract

**Introduction:**

maternal mortality is still a problem attracting global attention, with an estimate of 289,000 maternal deaths annually. Over half of these deaths occur in sub-Saharan Africa, with Nigeria accounting for 14% of the global maternal deaths. This study assessed the trends and patterns of maternal deaths, associated factors, and pregnancy outcomes in a rural area in Lagos, Nigeria

**Methods:**

this study adopted a cross-sectional descriptive research design. A retrospective assessment of all maternal deaths that occurred at Epe Local Government Area (LGA) from 2015 to 2019 was done. A validated checklist was used to retrieve information from the records of 96 deceased. Ethical approval was obtained for the study. Data collected were analyzed using SPSS version 20.0. Analyses were done using descriptive and inferential statistics at a significance level of 0.05.

**Results:**

highest number of maternal deaths 24 (23%) were recorded in the year 2015. The maternal mortality ratio was 1,645 per 100,000 live births. The highest direct cause of maternal death was eclampsia (27.1%), while the highest indirect cause was anemia (5.2%). Chances of maternal death increased with nonregistered pregnancy (71.9%), and non-institutional delivery (79.2%). Poor pregnancy outcomes include; stillbirth (60.4%), and preterm babies (62.5%). Statistical associations were found between maternal deaths and parity (p = 0.004).

**Conclusion:**

the maternal mortality ratio in rural areas is still very high and far from the proposed global target of 70 per 100,000. These maternal deaths are linked to direct and indirect causes. Maternal deaths could result in poor pregnancy outcomes.

## Introduction

Every day around the world, approximately 830 women die from preventable causes related to pregnancy and childbirth. Most of the deaths (99%) occur in developing countries [1]. It was estimated that in 2015, roughly 303,000 women died during and following pregnancy and childbirth. Almost all of these deaths occurred in low-resource settings, and most could have been prevented [1]. World Health Organization [2] defines maternal mortality as the death of a woman while pregnant or within 42 days of termination of pregnancy irrespective of the duration and site of the pregnancy, from any cause related to or aggravated by the pregnancy or its management but not from accidental or incidental causes [3], opined that in sub-Saharan Africa, a number of countries halved their levels of maternal mortality since 1990. Pregnancy and the period surrounding it is a dangerous time for too many of the 9.2 million women and girls who become pregnant in Nigeria each year. They face a lifetime risk of maternal death of 1 in 13 compared to 1 in 31 for sub-Saharan Africa as a whole [4]. One target under the third Sustainable Development Goal (SDG 3) is to reduce the global maternal mortality ratio to less than 70 per 100 000 births, with no country having a maternal mortality rate of more than twice the global average [2]. The associated risk of maternal mortality is higher in women living in rural areas and in poorer communities [5]. The range is wide and includes the behavior of families and communities, social status, education, income, age, parity, booking status, place of birth, and availability of health services [6]. Higher risks of death have been associated with no formal education, primary education only, lack of antenatal and postnatal care, and cesarean section births [7]. Determinants of deaths were also defined as individual risk factors, such as age and parity; characteristics of the social, legal, and economic contexts; and the physical environment, for example, geographical accessibility (rural or urban) [7].

In Nigeria, Maternal Mortality Ratio (MMR) had initially risen from 704 per 100,000 live births in 2000 to 800 in 2003 before a gradual reduction to 545 in 2007 with an increased rate of 814 per 100,000 live births in 2015 [2,8]. Therefore, there is so much work to be done in curtailing maternal deaths; this has been supported by relevant stakeholders leading to the current Sustainable Development Goals (SDG) target of achieving less than 70 maternal deaths per 100,000 live births by 2030, as a consolidation of the gains of the MDG achievements [9]. Women die of several complications during the perinatal, intrapartum, and postpartum periods. Most complications develop because of pregnancy, (direct causes), and some because pregnancy aggravated existing diseases (indirect causes) such as gestational diabetes and pregnancy induced hypertension. The five major direct causes of maternal death as identified by the World Health Organization are Post-Partum Hemorrhage (PPH), Puerperal sepsis, Hypertensive disorders in pregnancy (Pre-eclampsia and Eclampsia), obstructed labor, and complications following unsafe abortion [2]. Pregnancy-related deaths are still unacceptably high in many low-income countries, where pregnancy and childbirth are high-risk events that constitute major public health challenges. At 576 maternal deaths per 100,000 live births, Nigeria accounts for roughly 14 percent of the global burden of maternal mortality [10,11]. The prevalence of PPH, sepsis, eclampsia and unsafe abortion in Nigeria is 4.5%, 9.2%, 1.7% and 1.5% respectively [12,13].

In many developing countries, a mother´s death is much more than an emotional crisis. The death of a mother often leads to long-term economic and social breakdown, both for her immediate family, surviving children, households, community, and the nation at large [14]. When a mother dies from causes related to pregnancy and childbirth, the consequences are interlinked, intergenerational, and extensive. Studies have shown increased mortality among children whose mothers died during pregnancy and childbirth [14,15]. A study by Israel M *et al*. [16] revealed that newborns whose mothers die in childbirth are far less likely to reach their first birthday than those whose mothers do not die. However, most of the studies conducted on causes of maternal deaths did not communicate research findings back to the settings where such were conducted. Also, most studies on maternal death in Lagos state focused more on the major referral centers which are mostly in the interior/urban region of the state, neglecting the exterior/rural region such as Epe. There are limited studies done to explore the causes and associated factors of maternal deaths in the selected local government area (Epe). Hence, this study examined the trends, patterns, and factors associated with maternal deaths as well as the pregnancy outcomes of maternal deaths since the commencement of SDGs.

## Methods

**Study design:** this study adopted a descriptive cross-sectional research design, using a quantitative approach, and a retrospective method.

**Study setting:** Epe Local Government area of Lagos state, Nigeria was selected for this study. This is one of the rural areas of Lagos state.

**Study population:** the study population included women who died as a result of pregnancy or within 42 days of termination of pregnancy or its management in Epe Local Government within the study period of the year 2015 to 2019. This was to ensure the most recent information on maternal deaths is captured and to also analyze the death rate in Epe local government since the adoption of Sustainable Development Goals (SDGs) by the global community. All maternal deaths that occurred during the study period were identified from the maternal mortality registers and Lagos State monthly returns of the hospitals and health facilities.

**Sampling techniques:** a purposive sampling procedure was used to select a rural health facility that has at least one maternal death among the eighteen (18) Primary Health Centers and the two (2) General Hospitals in Epe local government area within the study period of 2015 to 2019. The records of 96 women who died as a result of pregnancy or within 42 days of termination of pregnancy within the study period were extracted from the registry. The maternal mortality rate was calculated by dividing the total number of maternal deaths during the study period by the total number of live births during the study period.

**Instrument/data collection:** a validated structured checklist developed by the researchers was used as a guide to elicit information from the deceased medical records. The records of all maternal deaths that met the inclusion criteria were obtained from the appropriate units of the hospital, and relevant information/case abstraction was done.

**Data analysis:** data were entered into the Statistical Package for Social Sciences, SPSS version 20.0. Descriptive statistics such as frequency distribution tables, charts, and figures were used to analyze and summarize the data collected. The results from the frequency distribution table were interpreted in percentages, and multivariable logistic regression was used. The Chi-square test was used to test for association between variables. The significance level was determined at P <0.05. Data were analyzed based on the set objectives and hypotheses of the study.

**Ethical approval:** ethical approval to conduct the research was obtained from the Nigerian Institute of Medical Research Ethical Review Board, Yaba Lagos State. Confidentiality was maintained by not including names and addresses of the deceased. Information collected was treated with anonymity. No form of identifier was included in the checklist, instead, code numbers were used. Information elicited from the participants´ records was not disclosed except for research purposes.

## Results

**Socio-demographic characteristics of the participants:** a total of 96 maternal case files of women aged 15 to above 45 were reviewed. Table 1 provides the demographic data of the participants of the study. Most deaths occur among those age 25 to 29 years, accounting for 34 (35.4%) cases with an average age of 28 years. Moreover, we see that in the parity of the women, more of the women (56) were multiparous with 3 children and above. Additionally, it is evident that the majority of the women (31 (32.3%)) were artisans, followed by businesswomen (26 (27.1%)), and housewives (22 (22.9%)).

**Table 1:** sociodemographic characteristics of women who died in relation to pregnancy and childbirth in rural Lagos from 2015 to 2019

Socio-demographic characteristics	Frequency (n=96)	Percentage (%)
**Age**		
15-19	2	2.1
20 – 24	14	14.6
25 - 29	34	35.4
30 - 34	20	20.8
35 - 39	20	20.8
40 - 44	1	1.0
45 and above	5	5.2
**Parity**		
Primipara	12	12.5
Multipara	56	58.3
Grand multipara	28	29.1
**Occupation**		
House wife	22	22.9
Student	10	10.4
Business	26	27.1
Civil servant	7	7.3
Artisan	31	32.3
**Religion**		
Islam	55	57.3
Christianity	41	42.7
**Tribe**		
Yoruba	61	63.5
Igbo	26	27.1
Hausa	9	9.4

**Figure 1:**
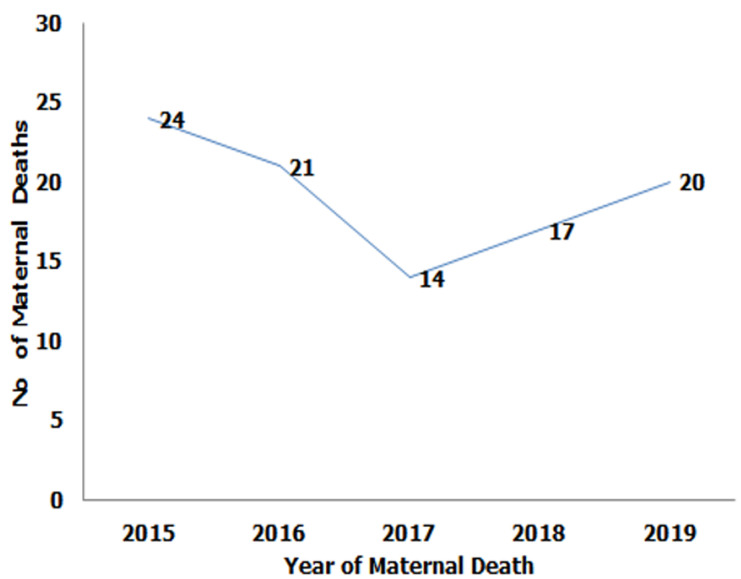
maternal deaths by year in Epe Local Government Area from 2015 to 2019

**Trends of maternal deaths in Epe LGA of Lagos state:** the year 2015 recorded the highest number of deaths, 24 (23%). In 2017, there was a sharp reduction in the number of maternal deaths to 14 (13.4%). The figure increased again in 2019 to 20 maternal death cases (Figure 1).

**Maternal mortality ratio of Epe Local Government over the study period (2015 to 2019):** the calculated maternal mortality ratio for Epe Local Government was 1,645 per 100,000 live births. This implies that for every 100,000 births, there was an equivalent 1,645 maternal deaths.

**Figure 2:**
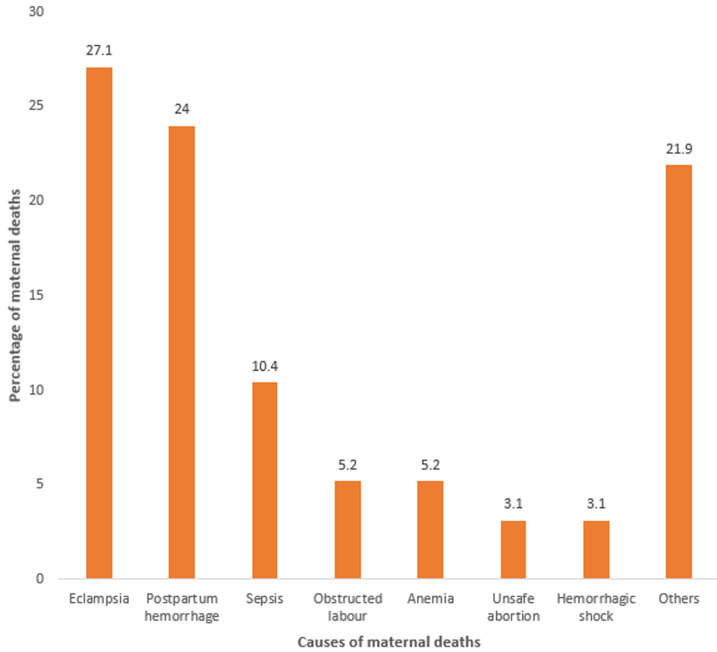
causes of maternal deaths in Epe Local Government from 2015 to 2019

**Causes of maternal deaths among participants:** the various causes of maternal deaths are illustrated in Figure 2. From the bar chart, it is crystal clear that the three highest direct causes of maternal death in descending order include eclampsia, 26 (27.1%), postpartum hemorrhage (PPH), 23 (24.0%), and sepsis 10 (10.4%). The two highest indirect causes of maternal death recorded was anemia, 5 (5.2%). A total of 21 (21.9%) of maternal deaths were from other causes which include; obstructed labor, unsafe abortion, ectopic gestation, placental previa, placenta abruption, ruptured uterus, septic shock, hemorrhagic shock, hypovolemic shock, disseminated intravascular coagulation (DIC), blood transfusion reaction and pulmonary embolism.

**Factors associated with maternal deaths among participants:** Table 2 shows the multinomial logistics regression and the odds ratio of the various factors that significantly influence maternal deaths. It can be inferred that the chances of maternal death increase with un-booked mothers, place of delivery outside a hospital, vaginal mode of delivery, and presence of comorbidities.

**Table 2:** multinomial logistics regression analysis of factors influencing maternal death

Factors	Maternal death N (%)	P-value	aOR(95%CI)
**Booking status**		<0.001	
Booked	27(28.1)		1
Un-booked	69(71.9)		0.36 (0.21 – 0.64)
**Site of birth**		0.001	
Hospital	20(20.8)		1
Home birth	35(36.5)		0.43 (0.38 – 5.39)
Traditional birth attendant	31(32.3)		0.54 (0.64 – 3.72)
Mission home	7(7.3)		0.78 (0.81 – 3.90)
**Mode of delivery**		0.443	
Vaginal delivery	75(78.1)		1
Caesarian section	13(13.5)		0.41 (0.16 – 1.02)
Assisted delivery	5(5.2)		0.77 (0.36 – 1.67)
**Presence of tears and lacerations during labor**		0.999	
Yes	19(19.8)		1
No	65(67.7)		1.55 (0.79 – 3.04)
Unknown	9(9.4)		2.47 (1.06 – 5.72)
**Stage of complication development**		1.112	
During pregnancy	31(32.3)		1
During labor/delivery	30(31.3)		0.22 (0.78 – 4.62)
After delivery	35(36.5)		0.37 (0.43 – 2.14)
**Clinical status on presentation**		1.332	
Conscious	36(37.5)		1
Semiconscious	32(33.3)		0.56 (0.72 – 3.27)
Unconscious	28(29.2)		0.88 (0.63 – 6.23)
**Presence of comorbidity**		<0.001	
Present	11(11.5)		1
Not present	16(16.7)		0.51 (0.45 – 3.57)
Unknown	69(71.9)		0.82 (0.62 – 2.17)
**Location at the point of complication development**		1.449	
Outside healthcare facility (referred cases)	92(95.8)		1
Healthcare facility	4(4.2)		0.28 (0.34 – 3.36)

**Proportion of referral cases among participants:** a majority (96%) of maternal deaths were as a result of complications occurring outside the healthcare facility. Such cases were referred to the hospital. Only 4% of maternal deaths were from in-patients.

**Pregnancy outcome of maternal death among the participants:** all the babies were either preterm 60 (62.5%) or small for their gestational age 30 (31.3%). Just small portions (30 (31.3%)) of the babies were born alive; majority of them were stillbirths (58 (60.4%)). Also, all the babies were born with mild to severe birth asphyxia (Table 3). The results of cross-tabulations using Chi-square show that parity (X^2^= 14.735, P = 0.004), antenatal registration or booking status of a woman (X^2^= 18.735, and P = 0.001), place of delivery (X^2^= 24.15, and P = 0.002) are statistically associated with maternal deaths.

**Table 3:** pregnancy outcomes of maternal deaths in Epe Local Government from 2015 to 2019

Pregnancy outcomes	Frequency (n)	Percentage (%)
**Was the baby delivered?**		
Yes	93	96.9
No	3	3.1
**What was the outcome of the pregnancy?**		
Live birth	30	31.3
Stillbirth/Intrauterine fetal death	58	60.4
Abortion	5	5.2
**Baby status at birth?**		
Termed	3	3.1
Preterm	60	62.5
Small for gestational age	30	31.3
Postdated	0	0.0

## Discussion

This study has delved into the trends of maternal deaths, patterns of maternal deaths, factors associated with maternal deaths, and pregnancy outcomes of maternal deaths over a period of five years (2015 to 2019). The MMR of Epe LGA during the study period was found to be very high. This among other things could be attributed to the very low utilization of health facilities for childbirth, which resulted in non-institutional delivery. Only one-fifth of the participants had institutional delivery. Also, only about one-quarter of the participants registered for antenatal care or were booked cases of the facility while the majority of the participants were referred from mission homes, traditional birth attendant (TBA) centers, and even home deliveries. The majority of these referrals wouldn´t have been done timely, resulting in late presentation of complications during labor. This is consistent with the WHO [17] assumption that the majority of maternal death is as a result of complications developed during pregnancy and childbirth, most of which are preventable or treatable. The high MMR in this study is similar to another study [18] conducted in Lagos eight years before the current study. This could imply that little attention is being paid to maternal deaths in rural areas. Therefore, effective interventions targeting the reduction of maternal mortality in rural areas should be given priority if SDG 3 will be achieved in Nigeria.

Furthermore, this study identified the various direct and indirect causes of maternal deaths, the three highest direct causes in descending order include; eclampsia, postpartum hemorrhage, and sepsis. This is also consistent with WHO´s assertions [17], and other studies conducted in Nigeria [19-22], and Zimbabwe [23]. This study also identified anemia as one of the most significant indirect causes of maternal deaths. This is supported by the report of Say *et al*. [3], who reported a systematic analysis by WHO. According to Say *et al*. [3], anemia, was identified as one of the factors responsible for more than half of maternal deaths worldwide. Also, Solwayo´s report [23] agreed with the identified direct and indirect causes of maternal deaths identified in this current study. These identified causes of death in this study may not be unconnected with non-institutional deliveries (home and TBA births) and delay in seeking care. Therefore, since many authors have identified these direct and indirect causes of maternal deaths over time, efforts should be harnessed among stakeholders in maternal health to provide definite solutions to the identified causes to reduce maternal deaths, especially in low and middle-income countries like Nigeria.

In addition, this study identified factors that increased the chance of maternal deaths. The odds of maternal death increased with women who did not register for antenatal care with a skilled provider or unbooked mothers, non-institutional delivery, and the presence of comorbidities. Moreso, more than three-quarters of the total maternal deaths recorded in this study were from unbooked mothers, delivery at a TBA center, and home delivery. These identified factors were similar to factors identified by Ezugwu *et al*. [24], and Faduyile *et al*. [25] in different studies conducted in Nigeria. The authors reported booking status and place of delivery as important interconnected factors which may significantly impact the chances of maternal mortality. The result of this study is also supported by a study at Ifakara, Tanzania by Alfred *et al*. [26]. The authors identified place of delivery as a risk factor for maternal deaths. Meanwhile, Patrick *et al*. [27] in another study in the Gambia discovered that advancing maternal age has strongly been associated with maternal deaths. However, this contradicts the result of this current study which showed a greater number of deaths occurring among women aged 25 to 29 years. This variation could be due to younger women in the rural areas of Lagos, Nigeria. Moreso, the rural areas in Lagos Nigeria are like cities in other regions of the country and other countries as well. Also, some of the results on associated factors resulting in maternal deaths may be due to cultural norms and beliefs, and poor utilization of health centers for pregnancy care and delivery in Epe local government area.

The pregnancy outcomes of maternal death in this current study, which include birth asphyxia, preterm delivery, and stillbirths, could be due to complications following the maternal deaths. This is supported by Israel *et al*. [16], who reported that many of the direct and indirect causes of maternal deaths have a significant impact on the baby outcome. In addition, Gillon *et al*. [28] also agreed with these assertions. The authors opined that there is a direct link between neonatal deaths, stillbirths, intrauterine fetal death (IUFD), and some of the causes of maternal death. This study also agrees with a study by Harrison *et al*. [29], who established a relationship between stillbirths and maternal death. This finding is also in tandem with WHO´s report [2], which stated that eclampsia is one of the most common causes of adverse outcomes of pregnancy worldwide and has a close relationship with stillbirth deliveries. Pregnancy outcomes are associated with maternal deaths, which could be fallouts from various complications.

This study found a significant association between the parity of a woman and her chances of dying in childbirth. This assertion is supported by Tadele *et al*. [30] and Patrick *et al*. [27]. Tadele *et al*. in a review established an association between parity and maternal deaths. Also, Patrick *et al*. supported this assertion in another study conducted in the Gambia. Therefore, this could be a problem in developing countries, where a woman has four or more children. Thus, appropriate family planning interventions could prevent maternal deaths. In addition, this study established an association between place of delivery and maternal deaths. This is consistent with other studies conducted in Nigeria [31-34] and sub-Saharan Africa [35]. However, unplanned home delivery and TBA births may be attributed to low socioeconomic status, cultural norms, and poor knowledge of institutional delivery among pregnant women. Therefore, interventions to promote institutional delivery among rural women will be recommended for all stakeholders in maternal health. This current study found no link between maternal age and maternal deaths. However, this negates several other studies [26,31,36] that found maternal age as a significant influence on maternal deaths. This could be because the majority of the study participants in the current study are younger women. Hence, there is no balance proportion of younger and older women. Thus, the effects of advancing age on maternal deaths could not be properly identified in the study. This could be one of the limitations of the study.

**Limitations of the study:** during the period of data collection, some (7) of the mothers´ case files were missing, reducing the study population to 96. Some vital information about the patients was not documented, and this made the gathering of important information very challenging for the researcher. Documentation of some causes of maternal deaths was not done using the WHO ICDs codes. Also, some health information officers were not supportive enough during the retrieval of case notes. In addition to that, the PHCs in Epe local government are scattered across the 18 political wards, hence visiting those places was really challenging and stressful. Record keeping at the PHC level was not adequate.

## Conclusion

The research study was carried out to assess the trends of maternal deaths, identify patterns of maternal deaths, associated risk factors of maternal deaths, and the pregnancy outcome of maternal deaths in rural Lagos, Nigeria from 2015 to 2019. The study established that the highest maternal deaths occurred in the year 2015, and the three highest direct causes of maternal death in descending order include eclampsia, postpartum hemorrhage, and sepsis. Among the highest indirect causes of maternal death recorded are anemia, septic shock, Disseminated Intravascular Coagulation (DIC), and pulmonary embolism. There is an increased chance of maternal death with non-registration for antenatal care or unbooked mothers, and non-institutional delivery. Also, parity and place of delivery could have a significant impact on maternal deaths.

### What is known about this topic

- *Maternal mortality is a public health problem receiving global attention, especially in developing countries including Nigeria;*
- *Maternal mortality is one of the indices for assessing the development of any nation;*
- *Maternal deaths could be linked to both direct and indirect causes*.

### What this study adds

- *This current study established the trends of maternal deaths over a period of five years; the highest maternal deaths occurred in the year 2015, the same year the achievement of Millennium Development Goals (MDGs) assessment ended*;
- *The highest direct cause of maternal deaths was identified as eclampsia, while the highest indirect cause was identified as anemia; this provides information on planning appropriate interventions to reduce maternal deaths*;
- *The chance of maternal deaths increases with non-registration for antenatal care with skilled care providers, and non-institutional delivery; this information serves as basis for provision of adequate awareness program that will promote antenatal care registration and institutional delivery*.

## References

[1] Alkema L, Chou D, Hogan D, Zhang S, Moller AB, Gemmill A (2016). Global and National Levels and Trends in Maternal Mortality; A systematic analysis by the United Nation Maternal Mortality Estimate Agency. Lancet.

[2] World Health Organization (2016). World health statistics: monitoring health for the SDGs.

[3] Say L, Chou D, Gemmill A, Tuncalp O, Moller AB, Daniel J (2014). Global Causes of Maternal Death. A WHO Systematic Analysis. Lancet Glob Health.

[4] Ganatra B, Gerdts C, Rossier C, Johnson J, Tuncalp O, Sedgh G (2017). Global, regional, and subregional classification of abortions by safety, 2010-14: estimates from a Bayesian hierarchical model. Lancet.

[5] Oye-Adeniran BA, Odeyemi KA, Gbadegesin A, Ekanem EE, Osilaja OK, Akin-Adenekan O (2011). The use of the sisterhood method for estimating maternal mortality ratio in Lagos state, Nigeria. J Obstet Gynaecol.

[6] Rogo K, Oucho J, Mwalali P, Jamison DT, Feachem RG, Makgoba MW, Bos ER, Baingana FK, Hofman KJ, Rogo KO (2006). Maternal Mortality. Disease and Mortality in Sub-Saharan Africa.

[7] Bauserman M, Lokangaka A, Thorsten V, Tshefu A, Goudar SS, Esamai F (2015). Risk Factors for Maternal Death and Trends in Maternal Mortality in low and middle-income countries; A Prospective Longitudinal Cohort Analysis. Reproductive Health Journal.

[8] Ishola LA, Frank A, Leke BK (2015). Can Nigeria Achieve Millennium Development Goals. The Journal of Social Sciences Research.

[9] World Health Organization (2015). Strategies Towards Ending Preventable Maternal Mortality (EPMM). WHO Bulletin.

[10] Kassebaum JN, Bertozzi-Villa S, Coggeshall M, Steiner T, Heuton C (2014). Global, Regional and National Levels and Causes of Maternal Mortality; A Systematic Analysis for the Global Burden of Disease Study. Lancet.

[11] World Health Organization (2014). Maternal Mortality Fact Sheet. World Health Organization.

[12] Ononge S, Mirembe F, Wandabwa J, Campbell OM (2016). Incidence and Risk Factors for Postpartum Hemorrhage in Uganda. Reprod Health.

[13] John S, Sumedha JI, Tang L, Marianne V, Akinmade I, Adepoju AA (2016). The Hypertensive Disorders of Pregnancy in Ogun State, Nigeria. An International Journal of Women's Cardiovascular Health.

[14] Emily JB, Jones R, Leslie B, Vanessa B, Emily M, Pilira K (2015). Intergenerational impacts of maternal mortality: Qualitative findings from rural Malawi. Reproductive Health Journal.

[15] World Health Organization (2015). Maternal Mortality Fact Sheet.

[16] Israel M, Mitiku M, Alemayehu W, Alicia E (2015). Impact of Maternal Mortality on Living Children and Families. Reprod Health.

[17] World Health Organization WHO updates fact sheet on Maternal Mortality (19 September 2019).

[18] Okonofua FE (2010). Reducing Maternal Mortality in Nigeria. An Approach through Policy Research and Capacity Building. African Journal of Reproductive Health Sept.

[19] Ujah AO, Aisien OA, Mutihir JT, Glew RH, Uguru (2005). Factors Contributing to Maternal Mortality in North Central Nigeria. Afr J Reprod Health.

[20] Nwobodo Y, Ahmed (2011). Maternal Mortality Associated with Eclampsia in Sokoto, Nigeria. Orient Journal of Medicine.

[21] Babah OA, Oluwole AA, Afolabi BB, Odum CU (2014). A Review of Eclampsia at Lagos University Teaching Hospital (LUTH). Journal of Dental and Medical Sciences.

[22] Ouonuju CN, Nyeugidiki TK, Ugboma HA, Bassey G (2015). Risk Factors and Antibiogram of Organisms Causing Puerperal Sepsis in a Tertiary Health Facility in Nigeria. Tropical Journal of Obstetrics & Gynaecology.

[23] Solwayo N (2016). Postpartum Hemorrhage; Incidence, Risk Factors, and Outcomes in a Low-Resource Setting. International Journal of Women´s Health.

[24] Ezugwu EC, Agu PU, Nwoke MO, Ezugwu FO (2014). Reducing Maternal Deaths in a Low Resource Setting in Nigeria. Niger J Clin Pract.

[25] Faduyile FA, Soyemi SS, Emiogun FE, Obafunwa JO (2017). A 10 years Autopsy-Based Study of Maternal Mortality in Lagos State University Teaching Hospital, Lagos, Nigeria. Niger J Clin Pract.

[26] Alfred K, Nathan R, Nelson G (2018). Maternal Mortality in Ifakara Health and Demographic Surveillance System; Spatial Patterns, Trends and Risk Factors. PLoS One.

[27] Patrick I, Matthew O, Anyanwu E, Sabel B (2017). A Retrospective Analysis of Trends in Maternal Mortality in a Gambian Tertiary Health Centre. BMC Res Notes.

[28] Gillon TE, Pels A, Von P, MacDonell K, Magee LA (2014). Hypertensive Disorders of Pregnancy; A Systematic Review. PLoS One.

[29] Harrison MS, Ali A, Pasha O, Salem S, Althabe F (2015). A Prospective Population-Based Study of Maternal, Fetal, and Neonatal Outcomes in the Setting of Prolonged Labor, Obstructed Labour and Failure to Progress. Reprod Health.

[30] Girum T, Wasie A (2017). Correlates of Maternal Mortality in Developing Countries; A Study in 82 countries. Maternal Health, Neonatology and Perinatology.

[31] Okonofua F, Imosemi D, Igboin B, Adeyemi A, Chibuko C, Idowu A (2017). Maternal Death Review and Outcomes; An assessment in Lagos State, Nigeria. PLoS One.

[32] Sageer R, Kongnyuy E, Adebimpe WO (2019). Causes and Contributory Factors of Maternal Mortality; Evidence from Maternal and Perinatal Death Surveillance and Response in Ogun State, Southwest Nigeria. BMC Pregnancy Childbirth.

[33] Akinwaare MO (2014). Availability of skilled birth attendance during home delivery among postpartum women in Ibadan, Nigeria. African Journal of Nursing and Health issues.

[34] Akinwaare MO, Adejumo PO (2015). Determinant of Choice of Place of Birth and Skilled Birth Attendant among Childbearing Women in Ibadan, Nigeria. African Journal of Midwifery and Women´s Health.

[35] Chinkhuma J, De-Allegri M, Muula AS (2014). Maternal and Perinatal Mortality by Place of Delivery in Sub-Saharan Africa; A Meta-analysis of Population-based Cohort Studies. BMC Public Health.

[36] Olajide DO, Akinwaare MO, Ede NA (2021). Pregnancy Outcomes and Associated Factors among Older Women in Adeoyo Maternity Teaching Hospital, Ibadan, Nigeria. International Journal of Caring Sciences.

